# Mapping nutrient resorption efficiencies of subarctic cryptogams and seed plants onto the Tree of Life

**DOI:** 10.1002/ece3.1079

**Published:** 2014-05-07

**Authors:** Simone I Lang, Rien Aerts, Richard S P van Logtestijn, Wenka Schweikert, Thorsten Klahn, Helen M Quested, Jurgen R van Hal, Johannes H C Cornelissen

**Affiliations:** 1 Systems Ecology, Department of Ecological Science, VU UniversityDe Boelelaan 1085, 1081 HV, Amsterdam, The Netherlands; 2 State Museum of Natural History KarlsruheErbprinzenstr. 13, 76133, Karlsruhe, Germany; 3 Fraunhofer Institute for Chemical Technology (ICT)Pfinztal-Berghausen, Germany; 4 Department of Animal and Plant Sciences, The University of SheffieldSheffield, U.K

**Keywords:** Bryophyte, conducting tissue, evolutionary specialization, internal nutrient cycling, lichen, phylogeny, pteridophyte, senescence, translocation, vascular plant

## Abstract

Nutrient resorption from senescing photosynthetic organs is a powerful mechanism for conserving nitrogen (N) and phosphorus (P) in infertile environments. Evolution has resulted in enhanced differentiation of conducting tissues to facilitate transport of photosynthate to other plant parts, ultimately leading to phloem. Such tissues may also serve to translocate N and P to other plant parts upon their senescence. Therefore, we hypothesize that nutrient resorption efficiency (RE, % of nutrient pool exported) should correspond with the degree of specialization of these conducting tissues across the autotrophic branches of the Tree of Life. To test this hypothesis, we had to compare members of different plant clades and lichens within a climatic region, to minimize confounding effects of climatic drivers on nutrient resorption. Thus, we compared RE among wide-ranging basal clades from the principally N-limited subarctic region, employing a novel method to correct for mass loss during senescence. Even with the limited numbers of species available for certain clades in this region, we found some consistent patterns. Mosses, lichens, and lycophytes generally showed low RE_N_ (<20%), liverworts and conifers intermediate (40%) and monilophytes, eudicots, and monocots high (>70%). RE_P_ appeared higher in eudicots and liverworts than in mosses. Within mosses, taxa with more efficient conductance also showed higher RE_N_. The differences in RE_N_ among clades broadly matched the degree of specialization of conducting tissues. This novel mapping of a physiological process onto the Tree of Life broadly supports the idea that the evolution of conducting tissues toward specialized phloem has aided land plants to optimize their internal nitrogen recycling. The generality of evolutionary lines in conducting tissues and nutrient resorption efficiency needs to be tested across different floras in different climatic regions with different levels of N versus P availability.

## Introduction

Plant adaptations to nutrient-poor environments include low-nutrient requirements of plant tissues and high tissue longevity together with high resorption of nutrients from senescing parts (Chapin 1980; Reich et al. 1992; Aerts 1995; Killingbeck 1996). Although resorption of nutrients, especially nitrogen (N) and phosphorus (P), is a well-known process from higher plants (Aerts 1996; Killingbeck 1996; Freschet et al. 2010; Vergutz et al. 2012), the controlling factors in nutrient resorption efficiency (RE) have remained elusive. While large differences in leaf-nutrient resorption have been found among species, differences in RE between vascular plant growth forms appear inconsistent and not large on average (Aerts 1996; Killingbeck 1996; Yuan and Chen 2009; Vergutz et al. 2012). Some consistent variation in nutrient RE correlated with deeper taxonomical position was reported by Killingbeck (1996), yet his study included a few seed plants only. Furthermore, environmental conditions have been suggested to influence intraspecific variation in RE (Killingbeck 1996; Lu et al. 2012); or they may have led to adaptation of whole-plant assemblages, as indirectly suggested by the large-scale increase in leaf N resorption efficiency (RE_N_) and the decrease in P resorption efficiency (RE_P_) of woody plants with latitude (Yuan and Chen 2009; Reed et al. 2012). The latter most likely reflects the predominant N deficiency of ecosystems at high latitudes, where soils are relatively young, as compared to P limitation in ancient soils, which predominate in the (sub)tropics (Lambers et al. 2008). While much work has been done on seed plants, other terrestrial autotrophs have been largely neglected. Among pteridophytes, only a few ferns have been studied for nutrient resorption (e.g., Killingbeck et al. 2002; Vergutz et al. 2012), while lycophytes and horsetails have been excluded almost entirely (but see Headley et al. 1985). Moreover, research on variation in and controls on nutrient resorption in cryptogams is still in its infancy (Cornelissen et al. 2007), even though bryophytes and lichens are paramount contributors to biomass, especially at higher latitudes where they fulfill important roles in nutrient and carbon cycling (Longton 1997; Cornelissen et al. 2007).

Here, we introduce a new concept to the debate about what controls nutrient resorption efficiency across taxa by proposing that species’ resorption efficiencies are determined importantly by evolutionary appearance of and changes in conducting tissues. Basic to this concept is that nutrients are translocated via the phloem during senescence (Gan 2007). Differences in conducting tissue should therefore importantly determine the extent of nutrient resorption. What do we know about tissue conductance of the main autotrophic, terrestrial clades of the Tree of Life? While nonvascular cryptogams contain no true sieve elements (SE; Behnke and Sjolund 1990), conducting tissue as such, albeit simple, have evolved in both liverworts and mosses (Hébant 1977). Phloem emerged in early cryptogams (lycophytes or club mosses, monilophytes or ferns, and horsetails) but was still relatively primitively built. In contrast, spermatophytes or seed plants (conifers, eudicots, and monocots) feature a differentiated phloem with sieve cells or tubes accompanied by specialized parenchyma cells (Behnke and Sjolund 1990). Thus, the development of conducting tissue during land plant evolution, from nonvascular cryptogams to tracheophytes (vascular plants), did not only help to bring about increasingly complex plant structures (Behnke and Sjolund 1990) but also efficient transport of a variety of compounds, such as photosynthates and amino acids, from leaves to other plant parts (Van Bel 2003). We propose that this development also must have offered increasing possibilities of internal nutrient recycling, especially N and P, from senescing photosynthetic tissues back to other plant parts, thereby helping the plants to gain relative independence from soil-nutrient status. In this paper, we ask the questions (1) whether the general lack or low degree of specialization of conducting tissues in nonvascular cryptogams compared with that in vascular plants has left them less efficient at nutrient resorption from senescing parts; and (2) whether interspecific variation within basal cryptogam clades corresponds with the presence/absence or degree of differentiation of conducting tissues as related to their phylogenetic position.

Thus, we hypothesize that the appearance and specialization of conducting tissues across the autotrophic branches of the Tree of Life has been accompanied by an evolution of increasing nutrient resorption efficiency. We test this new hypothesis across 16 lichen, 27 bryophyte, and 25 vascular plant species together comprising the predominant components of the subarctic bogs, mires, tundras, and forests of northern Europe, and covering the main basal clades of the Tree of Life present in a subarctic flora. With our comparative approach, we cannot account for phenotypic responses of nutrient resorption of individual species to nutrient availability in situ. However, we specifically chose to collect data from one climatic region, with broad prevalence of nutrient stress, thus avoiding strong confounding effects of gradients in climate and nutrient availability (see Yuan and Chen 2009). Also, we took care that the sampling environments were natural and typical for the species; the actual resorption efficiencies that we find are therefore more representative for the various higher taxa than if we had grown all species at similar but highly unrepresentative nutrient regimes. We apply a new methodology to allow fair, calibrated comparisons of mass loss–corrected nutrient resorption efficiencies among diverse taxa, by expressing nutrient pools of fresh and senesced tissues, respectively, relative to their contents of inert structural chemistry derived from infrared spectra (Fourier transform infrared–attenuated total reflectance (FTIR–ATR)). To our knowledge, this is possibly the first paper to link a physiological process, in this case nutrient resorption, explicitly to substantial branches of the plant phylogeny. It thereby makes a novel contribution to the newly proposed ambitious research agenda of mapping carbon and nutrient cycling onto the Tree of Life (Cornelissen and Cornwell 2014).

## Materials and Methods

### Sampling and species classification

**Figure 1:**
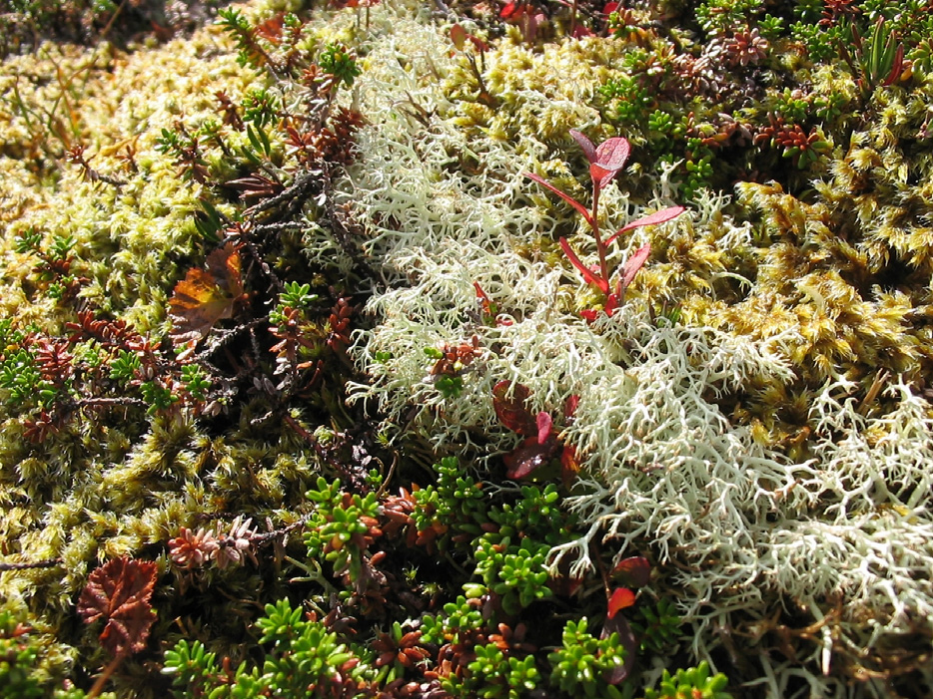
Vegetation on Andøya island, Norway, showing examples of subarctic species tested here for nutrient resorption efficiency in the Subarctic, as representatives of contrasting clades: mosses (*Racomitrium lanuginosum*), lichens (*Cladonia stygia*) and several vascular plant species (including *Empetrum nigrum*, *Vaccinium uliginosum*, *Rubus chamaemorus*, *Andromeda polifolia*). Photo by Marc Roβkopf.

Bryophytes and lichens (Fig. 1) were sampled in the summer of 2004 mainly around Abisko, Sweden (68°21′N, 18°49′E), but also on Andøya, Norway (69°07′N, 15°52′E) and in Kilpisjärvi, Finland (69°03′N, 20°50′E). The lichen *Cladonia stellaris* was sampled in the Altai Republic, S Siberia (51°04′N, 85°45′E) in 1999 and stored air-dry. We focused mainly on abundant species (see also Lang et al. 2009). We searched for patches large enough to allow for later nutrient analysis. Samples were taken where they were abundant, that is, where they showed optimal growth. At a given spot, several patches or several single specimens (depending on growth habit) were taken and pooled. For the vascular plants, we used an existing database, for which common species were sampled from the predominant ecosystems within 10 km from Abisko in 1998 and 1999 (Quested et al. 2003). As in the vascular plant dataset, no P was measured, and we took RE_P_ for six eudicots in the Abisko region from Van Heerwaarden et al. (2003b). Together these species were representative of the European subarctic region. For nomenclature see Lang et al. (2009).

Phylogeny followed Donoghue (2005). Species were allocated to basal clades, classes, orders, and families according to Stevens (2001 onwards) for vascular plants, Goffinet and Shaw (2009) for bryophytes and Lumbsch and Huhndorf (2007) for lichens (for the full list see Appendix S1). Not all cryptogam classes and orders could be represented by sufficient numbers of species, reflecting their low species richness in the European subarctic flora or the rarity of their occurrence. However, we feel that this imbalance, somewhat constraining detailed statistical analyses at the finer taxonomic levels, should still be acceptable compared with the disadvantages that would have been associated with adding species from other (climate) regions to artificially top up species numbers per group.

### Processing the cryptogam species

After return to the laboratory, samples were air-dried and kept in paper bags until further preparation. After careful remoistening without producing excess water to avoid leaching, cryptogams were thoroughly cleaned from dirt and other intermingled cryptogam species. Hereafter, liverworts and mosses were visually divided into the living green parts and the recently senesced (brown) parts (see Lang et al. 2009). Older, already visibly decomposed parts were not included. Similarly, lichens were divided into the living part and the recently senesced part, the latter with a seemingly softer structure, usually accompanied by a color change, that is, a dark brown, black or bleached appearance. For thallose lichens, senesced material was located in the center of the lichen.

In a second cryptogam dataset, we furthermore distinguished between early and late RE, as the green tissue in mosses often consists of several years’ growth (calculation see below). Mosses were visually divided into younger green tissue (bright green), older green tissue (darker green), and the recently senesced parts. Consequently, species that showed no differences in tissue color were excluded from this dataset, including all sampled liverworts and a few mosses. Younger versus older “green” tissue of all lichens was identified by its slightly green tinge (depending on thallus color) versus its mature thallus color, that is, brown or yellow.

**Figure 2:**
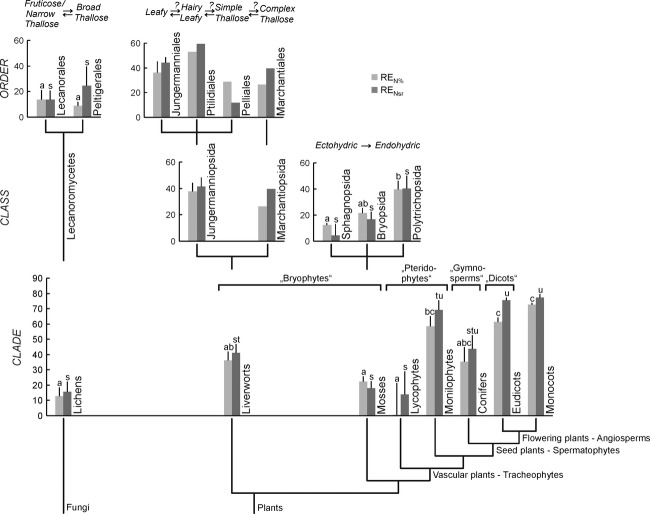
Nitrogen resorption efficiency (RE_N_) expressed as RE_N__%_ and RE_N__sr_ at clade, class and order level across autotrophic sections of the Tree of Life (lichenized fungi and plants). Different letters indicate significance at *P* < 0.05 (Tukey, *n* = 2–20), with a, b, c and s, t, u used for RE_N__%_ and RE_N__sr_, respectively.

The influence of choice of material on the magnitude of RE is illustrated in Appendix S2. In general (except for RE_P_ in lichens), RE was clearly higher in younger parts and lower in older tissue. Consequently, the measure of RE integrating all green tissue (Fig. 2), was 10–20% lower compared with RE in the youngest tissue. Given also the fact that mosses are known to move photosynthates both upwards into the shoot and downwards into senesced tissue as an energy store (Hakala and Sewón 1992), RE in cryptogams is dependent on the choice of material. In this study, we chose to use RE using all green tissue, in accordance with our sampling procedure for evergreen vascular cryptogams (lycophytes) where all green tissue comprising several years’ growth, is combined resulting in one RE value (Quested et al. 2003).

### Chemistry

Nitrogen concentrations of vascular plants were determined from ground samples, using a Tracermass mass spectrometer (Europa Scientific, Crewe, U.K.). For acid detergent fibers (ADF), cellulose and lignin analyses see Quested et al. (2003). P in vascular plants was determined colorimetrically at 880 nm with molybdenum blue (details see Van Heerwaarden et al. 2003b). The cryptogam samples, for which the following analyses were carried out, were ground for approximately 2 min using a ball mill (MM 200; Retsch, Haan, Germany) before use in further chemical analysis. For the concentrations of Ca and P, subsamples were acid digested (teflon bomb under addition of 1 mL of the mixture HNO_3_/HCl, ratio 4:1) for 7 h at 140°C. After adding 4 mL distilled water, Ca was measured by atomic absorption spectrometry (1100B Spectrometer; PerkinElmer Inc., Waltham, MA) under addition of 1% LaNO_3_. For P analyses see above. N was determined by dry combustion with a Carlo Erba NA1500 (Rodana, Italy) elemental analyzer. As cryptogam samples were cleaned meticulously, loss of ignition (mass LOI, at 550°C for 4 h) to correct for extraneous minerals, needed to be determined only for *Racomitrium fasciculare* and the lichen *Solorina crocea*. Both cryptogams originated from environments where contamination by minerals was possible. Molecular structure (i.e., oscillation of double bands in organic molecules) of the ground cryptogam and vascular plant samples was analyzed spectroscopically by FTIR-ATR (Nexus™ 670, ATR cell DuraScope; Thermo Nicolet, Madison, WI) with a resolution of 4 cm^−1^ and 32 scans. Extinction was calculated from infrared spectra followed by ground correction to correct for multiple scattering of light inside the probe. Further details of this methodology are in the study of Lang et al. (2009).

### Calculation and calibration of RE

Absolute nutrient concentrations of green versus senesced tissues might give incorrect mass-based RE (RE_%_) depending on the amount of translocation of carbon through plants or fungi (Van Heerwaarden et al. 2003a). Since for vascular plants, either area- (all except *Eriophorum vaginatum*) or leaf length-based RE_P_ (solely *E. vaginatum*) were available as a stable reference (Van Heerwaarden et al. 2003b), we combined these measures in the later analysis. We aimed to express nutrient pools based on an immobile fraction, such as total ADF, lignin, or cellulose. The latter occurs in vascular plants, bryophytes as well as in the algal part of lichens and can therefore be used as a stable reference for RE across clades. However, in most cryptogams, especially liverworts, the availability of material was too limited to perform the wet chemical laboratory analyses. Therefore, we conducted partial least squares regression (PLS-R; The Unscrambler v9.2; CAMO Software AS, Oslo, Norway) to identify ADF-, lignin- or cellulose-characteristic wavelengths using species where both wet chemical measurements and infrared measurements were available (*n* = 14; one moss, 13 vascular plants from contrasting clades). Based on significant variables only, which were determined with Jack-knifing (full cross validation), PLS-R was recalculated. In the final model, ADF and lignin were insufficiently described, while PLS-R for cellulose revealed an *R*^2^_Calibration_ of 0.98 and a small root mean square error_Calibration_ of 0.95. In a second step, we calculated cellulose based on wavelength for an independent set of species and compared the predicted cellulose values with conventional cellulose measurements. The linear relationship was significant (*P* = 0.003, *R*^2^ = 0.84). However, lichen cellulose content was not equally well expressed for all lichen species (details see Appendix S3). Calibration of lichen REs with Calcium (Ca) content (see Appendix S4), produced the same results for RE, both when compared to mass-based and cellulose-based RE, and the interaction term method x lichen order was not significant. We are therefore confident that our results are representative despite the above-mentioned difficulties with cellulose calibration for some lichen species.

Nitrogen RE_%_ (RE_N%_) was calculated as ([N_green_] − [N_senesced_])/[N_green_] × 100%, with N_green_ and N_senesced_ referring to N in green and senesced tissue, respectively. If calibrated with reference to immobile chemistry (for example, cellulose), RE_N_ with stable reference (RE_Nsr_) was expressed as ([N_green_]/[cellulose] − [N_senesced_]/[cellulose])/[N_green_]/[cellulose] × 100%. The corresponding parameters were calculated for P (RE_P%_ and RE_P_ with stable reference [RE_Psr_], respectively). Early and late RE were calculated as ([N or P_youngtissue_] − [N or P_oldertissue_])/[N or P_youngtissue_] × 100%, and ([N or P_oldertissue_] − [N or P_tissuesenesced_])/[N or P_oldertissue_] × 100%, respectively. For vascular plants, a complete dataset was solely available for green tissue in 1998 and for litter in 1999. We therefore compared [N_senesced_], and [N_senesced_]/[cellulose], of 1998 versus 1999, for species available in all datasets. Both linear regressions were highly significant, and, in the case of [N_senesced_]/[cellulose], the intercept was close to zero and the slope close to 1 (see Appendix S5). Thus, we concluded that differences in [N_senesced_] between years were relatively small, allowing a direct comparison of RE across adjacent years.

### Data analysis

Nitrogen RE of *Cetraria islandica* and RE_P_ of *Nephroma arcticum* and *Tomenthypnum nitens* were unrealistically very negative and strongly suspected to represent sampling or measurement problems. These outliers were excluded from further analysis. Where necessary, data were ranked to improve normality. The influence of taxonomic level, across basal clades and cryptogam orders and classes, on RE_%_ and RE_sr_ was tested in several one-way analyses of variance (ANOVAs) followed by Tukey's post hoc tests using SPSS 15.0 for Windows. The influence of method type on RE (i.e., RE_%_ vs. RE_sr_) was tested in a two-way ANOVA with method type and taxonomical level as between-subject factors. Within lichens, the influence of N-fixing ability on RE was tested in a one-way ANOVA. Where Levene's test remained significant despite data transformation, we chose to reduce sample size randomly down to five (or six) replicates (at basal clade level: RE_P_; testing method type and clade: RE_N_), as analysis of variance is robust to heterogeneity of variances as long as sample size is nearly equal (Zar 1999). Relating RE to [N_green_] in linear regression (y = ax + b) would violate the assumptions of independence in statistical tests. Therefore, we compared [N_senesced_] versus [N_green_] across clades and outlined the isoclines of RE_N%_ (0, 10, …90), as a function of [N_green_] and [N_senesced_], in the same graphs. With a positive slope, RE increases if the intercept b > 0 and decreases if b < 0. If b = 0, RE is constant across clades. We also compared N_senesced_/cellulose versus N_green_/cellulose to evaluate whether results deviated depending on the type of RE_N_ measure.

## Results

### RE_N_ and RE_P_

At a broad taxonomic scale, clade identity influenced both RE_N%_ and RE_Nsr_ significantly (Fig. 2, Table 1). Lichens (lichenized ascomycetes), mosses, and lycophytes showed lower RE_Nsr_ (and RE_N%_; <20%) compared with monilophytes, eudicots, and monocots (>70%), while liverworts held an intermediate position (40%). Conifer RE_Nsr_ and RE_N%_ did not differ significantly from other basal or derived clades. Clade was a significant determinant of RE_N_ (*F* = 23.72, *P* < 0.001; ranked), while method type (i.e., RE_N%_ vs. RE_Nsr_; *F* = 0.01, *P* = 0.93) and the interaction of clade x method type (*F* = 0.29, *P* = 0.95) were not significant. There was a consistent trend for differences in RE_P_ among clades. These differences are mainly due to the eudicots (RE_P%_ and RE_Psr_: 54 and 61%; or angiosperms: 60 and 66%) resorbing more P than mosses (32 and 28%), while lichens (17 and 20%), encompassing a wide data range, were not clearly separated from the other clades. RE_P_ in liverworts (42 and 50%) was almost as high as in eudicots.

**Table 1:** Statistical analysis of differences in nitrogen resorption efficiency (RE_N_) and phosphorus resorption efficiency (RE_P_) at clade, class and order level across the autotrophic sections of the Tree of Life (lichenized fungi and plants; *n* = 2–20). Significant *P*-values are marked with bold letters. Note that the underlying species sets are more robust for RE_N_ than for RE_P_ since RE_P_ of vascular plant clades encompassed solely eudicots (or angiosperms)

Clade	Taxonomic level	Source	d.f.	*F*	*P*
All	Clade	RE_N%_	7	15.03	**<0.001**
		RE_Nsr_	7	19.94	<0.001*
		RE_P%_	2	2.80† (2.62‡)	0.073*,† (0.079*,‡)
		RE_Psr_	2	2.88† (3.26‡)	0.068*,† (0.043*,‡)
Moss	Class	RE_N%_	2	3.98	**0.038**
		RE_Nsr_	2	2.79	0.089
		RE_P%_	2	0.78	0.47
		RE_Psr_	2	1.11	0.36
Lichen	Order	RE_N%_	1	0.10	0.76
		RE_Nsr_	1	0.45	0.51
		RE_P%_	1	5.48	0.036*
		RE_Psr_	1	3.37	0.089*

* Ranked.

† Eudicots.

‡ Angiosperms (eudicots and monocots).

At moss class level, the ectohydric Sphagnopsida showed lower RE_N%_ compared with the endohydric Polytrichopsida, while Bryopsida were intermediate. RE_Nsr_ showed a trend, showing the same pattern as RE_N%_. Class (*F* = 6.12, *P* = 0.005) was a significant determinant of RE_N_, while method type (*F* = 0.37, *P* = 0.55) and their interaction effect (*F* = 0.11, *P* = 0.90) were not significant. Neither RE_P%_ nor RE_Psr_ differed among moss classes.

At lichen order level, RE_N%_ or RE_Nsr_ of the fruticose and narrow thallose Lecanorales were not significantly different from the broad thallose Peltigerales. RE_P%_ was significantly higher in the Lecanorales and RE_Psr_ showed the same trend for these groups. N_2_-fixing lichens, which were present in both the Lecanorales and Peltigerales, did not show significantly lower RE_N_ (RE_N%_: *F* = 0.96, *P* = 0.35; RE_Nsr_: *F* = 0.19, *P* = 0.67), whereas RE_P_ was significantly higher in non-N_2_-fixing lichens (RE_P%_: *F* = 7.06, *P* = 0.020; RE_Psr_: *F* = 5.89, *P* = 0.031; ranked).

### The relation of N_senesced_ versus N_green_

**Figure 3:**
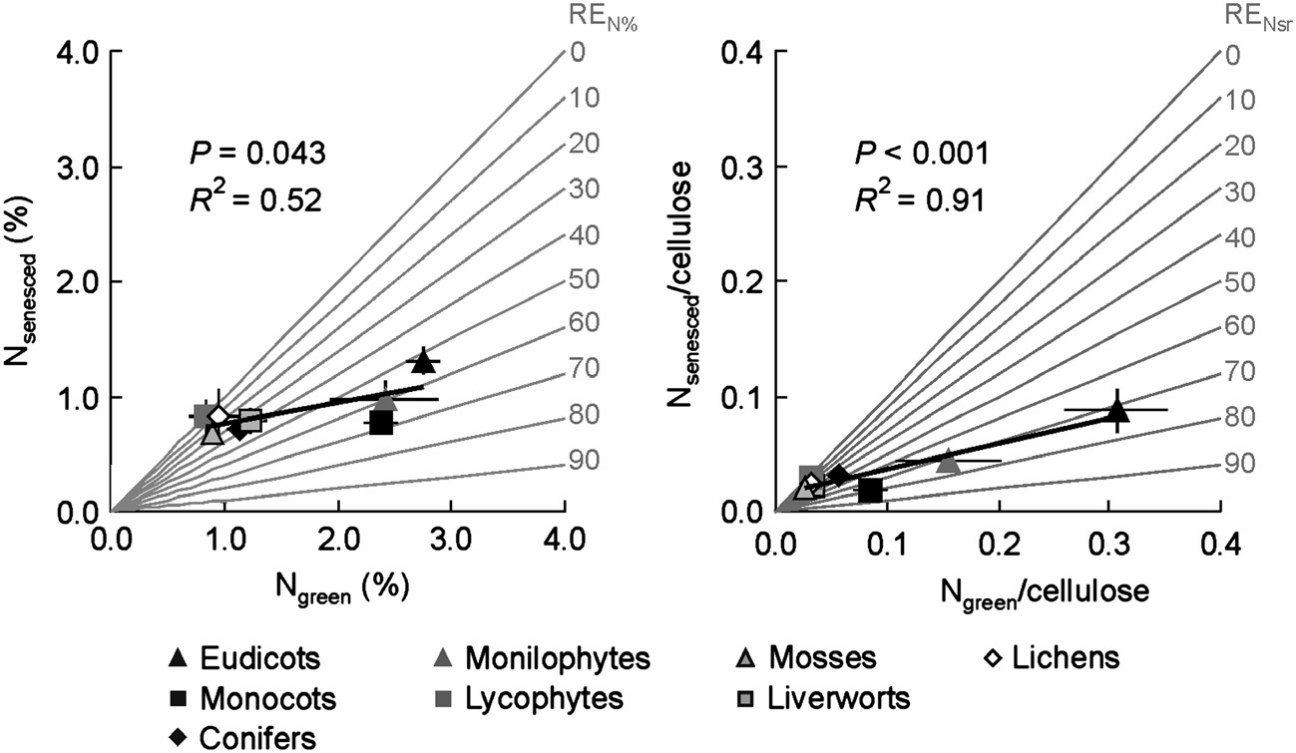
N_senesced_ (%) versus N_green_ (%), and N_senesced_/cellulose versus N_green_/cellulose, across the basal clades of the Tree of Life (±SE; *n* = 8). Isoclines for RE_N__%_ and RE_N__sr_ are outlined in gray.

Across clades, linear regressions between N_senesced_ and N_green_, whether based on percentage or cellulose, were positive and significant (Fig. 3). With increasing [N_green_] and [N_green_]/[cellulose], RE increased across clades (intercept of the regression line b > 0). Based on [N_green_] and even more so when looking at [N]/[cellulose], eudicots, monilophytes, and monocots showed the highest N_green_ content and highest RE_N_, while liverworts, conifers, mosses, lichens, and lycophytes were located at the lower end of the range.

## Discussion

All clades across the Tree of Life resorbed nutrients during organ senescence, but the efficiency differed strongly among clades. Mosses, lichens, and lycophytes generally showed low RE_N_, liverworts and conifers intermediate and monilophytes, eudicots, and monocots high. With reduced numbers of tracheophyte clades (only eudicots or angiosperms present), the pattern for RE_P_ was similar to RE_N_ but less clearly expressed. Within mosses, taxa with more efficient conductance also showed higher RE_N_. Thus, the variation in nitrogen resorption efficiency in a subarctic flora broadly supports the hypothesis that the evolution of conducting tissues has aided plants to optimize their internal nutrient cycling in terrestrial environments. While we have most confidence for our results for RE as expressed on a stable secondary chemistry basis, derived with our novel application of FTIR–ATR, the general correspondence of the between-clade patterns for RE_sr_ and RE_%_ show that the larger differences in RE are rather robust to methodological factors (interaction effect of clade x method type is not significant). Below we will discuss our findings in more detail with special focus on the types of conducting system that might support nutrient resorption of autotrophs across the Tree of Life.

### Conductive systems as vehicles for nutrient resorption across autotrophic clades

The fact that all nontracheophyte cryptogam clades had distinctly low RE_N_ compared to seed plants is consistent with the pattern of increasing differentiation of conducting tissue, from lichens, liverworts, and moss clades with no or little conducting tissue to the high differentiation of phloem in seed plants, although the apparently lower RE_N_ of conifers compared to angiosperms might be the results of other traits regulating RE (see below). Though based on fewer clades, the trend for RE_P_ was similar, with angiosperms showing highest RE_P_ in comparison with mosses, lichens and, to a lesser extent, liverworts. Our results for monilophytes and lycophytes were surprising, even though the numbers of species represented in this subarctic flora were too low for any firm statements. Still, the lycophytes showed low N resorption, while monilophytes had particularly efficient N resorption, even higher than the RE_N%_ of 52% reported for a temperate fern (Killingbeck et al. 2002). The phloem of lycophytes (e.g., *Lycopodium*) differs from other vascular cryptogams in possessing plasmalemma-lined sieve area pores which are wide open, whereas the pores of certain monilophytes, for example, *Equisetum* and leptosporangiate ferns, are traversed by membranes of endoplasmic reticulum (ER). In sieve tube members of eudicots, ER may play an important role in phloem-loading processes (Behnke and Sjolund 1990). However, recent studies suggest ER facilitates the trafficking of proteins (P-proteins in angiosperms, refractive spherules in monilophytes) between SE and companion cells (CC; Behnke and Sjolund 1990; Van Bel 2003). Such proteins, absent in conifers, lycophytes, and mosses, are thought to be involved in sugar metabolism, transmembrane sugar transport, membrane water permeability and protein degradation (Behnke and Sjolund 1990; Van Bel 2003). Thus, we speculate that the absence of P-proteins or refractive spherules in conifers and lycophytes may partly explain their low RE. In addition, the evergreen habit of both conifers and lycophytes might compensate for insufficient RE, conserving nutrients by extending their mean residence time (Aerts 1995). Furthermore, the low N concentrations in green tissue found in these clades (Fig. 3) limit the extent of RE (Aerts 1996), given that there is always a pool of N that remains immobile during senescence (Killingbeck 1996). We expected that the evolutionary emergence of specialized parenchyma cells, i.e., “Strasburger cells” in conifers and CC in angiosperms (Behnke and Sjolund 1990), would enhance the longitudinal flow in the phloem due to increased porosity of the end walls, which would then facilitate RE compared to that in vascular cryptogams (Van Bel 2003). However, RE of monilophytes was equally high as RE in seed plants, possibly owed to the presence of single cytoplasmatic connections between SE and parenchyma cells in this clade (Behnke and Sjolund 1990). It is tempting to assign differences in RE to minor vein type structure and their related phloem loading types, from ancestral symplastic phloem loading (ferns, conifers) to apoplastic or mixed loading in evolutionarily young angiosperms (Van Bel 2003). However, in the dicots, all three minor vein types are found (Turgeon et al. 2001) and, moreover, symplastic phloem loading also occurs in evolutionarily young angiosperms such as *Salix* (Van Bel 2003). Gamalei (1991) suggested evolutionary specialization of phloem loading in relation to climate. Typical in arctic-alpine tundra is the closed minor vein type, related to apoplastic phloem loading (except trees: symplastic) as symplastic plasmodesmal transport is sensitive to chilling. However, interactions between minor vein type and transported sugar identity on phloem loading type (Van Bel 2003), which is hypothesized to influence hydraulic gradient strength (Holbrook and Zwieniecki 2005), may further complicate the implications for resorption. Analogous to studies on xylem (Roth and Mosbrugger 1996), stelar morphology from a primitive protostele in the earliest land plants (e.g., *Cooksonia*, *Rhynia*) to eustele (e.g., eudicots), may have implications for phloem transport properties possibly allowing for increasing values of RE from primitive to sophisticated phloem transport properties.

In conclusion, many open questions remain concerning evolution of SEs and their cell biology, pathways and modes of phloem loading, impact of environmental factors on phloem transport and possible adaptation of phloem loading modes to climatic conditions (Van Bel 2003). Yet answering these questions will provide the basis from which we can start to evaluate the factors influencing RE in tracheophytes.

### Novel screening of nutrient resorption in higher cryptogam taxa

Our study is the first broad comparative study of nutrient resorption efficiency across and within basal cryptogam and seed plant clades. While for some bryophyte and lichen taxa, the subarctic flora did not support sufficient numbers of species for statistically sound comparison, we have quantified some consistent and logical patterns. Despite scattered evidence of N and P translocation for several moss species (e.g., Eckstein and Karlsson 1999), no study so far has investigated RE of a representative number of moss species in order to detect consistent taxonomic variation. Within mosses, translocation has been mainly linked to the endohydric Polytrichales (Collins and Oechel 1974; Reinhart and Thomas 1981) which feature leptoids (Hébant 1977), that is, conducting tissue, somewhat comparable with the sieve cells of higher plants. Also, this moss class alone features refractive spherules and callose, associated with plasmodesmata (Ligrone et al. 2000), which, in angiosperms, are associated with pores during sieve plate development (Behnke and Sjolund 1990). Indeed, RE_N%_ in the Polytrichaceae was higher than in other mosses, especially when compared to the ectohydric *Sphagnum*. However, though devoid of leptoids, transport of photoassimilates (Alpert 1989; Hakala and Sewón 1992) or N (Eckstein and Karlsson 1999) has also been reported for *Dicranum*, *Grimmia* and *Hylocomium*, respectively, most likely facilitated by an internal conducting strand of elongated parenchyma cells (Ligrone and Duckett 1994). Moreover, evidence has been found for conducting tissue in the Sphagnales differing from the Bryopsida only in lacking plastid – microtubules associations (Ligrone and Duckett 1998); this conducting tissue might explain P, C, and N translocation found for the peat moss *Sphagnum* (Rydin and Clymo 1989; Aldous 2002). We have to be aware that some interspecific variation in RE may be due to environmental factors. Furthermore, as not all mosses could be collected at the same time, species differences in seasonality of translocation (Skre et al. 1983), downwards for storage and upwards for growth, might have contributed to some of the observed variation. Also, cyanobacterial N fixation observed on feather mosses (Zackrisson et al. 2009) might have complicated the observed pattern. Indeed, RE_N_ of these mosses was higher than in most other species of the Bryopsida (data not shown).

This is, to our knowledge, the first study of RE of N and P in senescing photosynthetic parts of liverworts. With the exception of the complex thallose liverwort *Marchantia* (Rota and Maravolo 1975), little is known about translocation in this clade. Ligrone et al. (2000) suggested a microtubule-based translocation system for the marchantialean liverwort *Asterella*. However, liverworts seemed to generally resorb nutrients at rather high rates, comparable with those of conifers and monilophytes. A central strand of conducting tissue, which may lead to increased translocation of nutrients, has so far only been found for species in the liverwort orders Calobryales, Pallaviciniales (Hébant 1977), Pelliales and Marchantiales (Ligrone et al. 2000), of which the latter two were represented in our subarctic flora. The hypothesized link between central strands and RE is in need of explicit comparison across liverwort orders. Whether liverwort taxa representing different growth forms, (hairy) leafy, simple or complex thallose, show differences in RE, needs broader screening in other climatic regions as most species in this study belonged to the leafy liverwort order Jungermanniales, while all other orders were represented by only one (Ptilidiales) or very few species in the study area.

While several studies have reported nutrient resorption and translocation in nonsenescent tissues in lichens, nutrient resorption related to tissue senescence in lichens has hardly been studied at all. Translocation of N and P between fresh tissues has been demonstrated for *Cladonia stellaris*, *Stereocaulon paschale,* and *Cladonia portentosa*, respectively, (Hyvärinen and Crittenden 2000; Ellis et al. 2005; Kytöviita and Crittenden 2007), while for *Caloplaca trachyphylla*, transport of carbohydrates has been shown (Bench et al. 2002). Translocation in lichens, in which fungal hyphae provide the main structure, can most likely be compared with translocation in (ecto-) mycorrhizal mycelium. Out of three suggested transport mechanisms, diffusion, mass flow along turgor gradients or cytoplasmic streaming through pores of septa (Finlay 1992), the latter seems to be the most likely transport mechanism in lichens (Hyvärinen and Crittenden 2000). Assuming that a similar transport mechanism prevails in all fungal hyphae, we hypothesized that RE would not differ among lichens of different growth forms, when excluding N_2_-fixing lichens. Indeed, RE_N_ did not differ among lichen orders. However, RE_P%_ was higher in the Lecanorales and seemed to be negatively related to the N-fixing ability of lichens. The differences found in RE_P_ may be due to P in cyanobacteria. As cyanobacteria are located in cephalodia that are still present in dead lichen tissue, cyanobacterial P might not be accessible during resorption but is left behind in aging lichen tissue. Furthermore, algae in lichens are known to store P as polyphosphate in granules (Guschina et al. 2003), complicating the interpretation of the observed pattern. For N_2_-fixing lichens, with a constant input of readily available N, one would expect N resorption to be less important than in non-N_2_-fixing lichens, analogous to reduced RE_N_ of N_2_-fixing higher plants (Killingbeck 1996). However, N_2_-fixing lichens did not differ in RE_N_ from non-N_2_-fixing lichens, which may point to a similar transport mechanism across lichen species.

While in this study we concentrated on the relation of increasingly complex conducting tissue on RE, we acknowledge that other factors may also have important impacts on nutrient resorption. The influence of the environment on the poikilohydric bryophytes and lichens should not be underestimated since these cryptogams can take up nutrients via their whole surface. It is also known that nutrients can be lost from tissues through leaching. While this was shown to be of little significance to subarctic vascular plants (Freschet et al. 2010), we do not know the degree to which nutrients can be leached from different bryophytes and lichens. Also, the strength of the nutrient sink, for example, in roots of vascular plants, may be a factor contributing to nutrient resorption within the plants. Seasonality of translocation may play a role not only for mosses (Skre et al. 1983) but also for liverworts and lichens. Furthermore, while we know for a few moss species that nutrients can be stored in brown tissue (Skre et al. 1983), this has not been shown yet for liverworts and lichens. While all the above factors may have some influence on nutrient resorption efficiencies measured in broad species screenings, the fact that we found (mainly) coherent patterns among higher taxa, despite differences in sampling time and sampling from different environments, seems to strengthen the idea that conducting tissues indeed play an important role in nutrient resorption.

In conclusion, our results for an N-limited subarctic flora support the hypothesis that the progressive evolution of tissues to facilitate internal transport across the major clades of land plants is, by and large, coupled with their nutrient resorption efficiency during organ senescence. As such, this has led to a lesser dependency of plants on external nutrient supply. However, the generality of such evolutionary lines in conducting tissues and nutrient resorption efficiency needs to be tested more rigorously across different floras in different climatic regions with different levels of N versus P availability.

While many organism characters have been mapped explicitly onto the Tree of Life before, nutrient resorption may represent one of the first organismal *processes* to have been given this treatment. Under the assumption that actual nutrient resorption, which involves several interacting physiological and chemical processes (Gan 2007), is influenced by myriad genes (Gan 2007), our approach will be of great interest for phylogenetic analysis of other complex organismal processes as well.

## Data accessibility

The underlying data used for the analyses in this paper will be included upon publication as a digital database in Appendix S6.

## Supporting Information

**Appendix S1.** List of vascular plant and cryptogam species assessed for nutrient resorption efficiency. **Appendix S2.** Early versus late RE in lichens and mosses. **Appendix S3.** Prediction of cellulose content inferred from infrared spectra. **Appendix S4.** Comparison of RE calibration approaches. **Appendix S5.** Vascular plant [N_senesced_] and [N_senesced_]/[cellulose] of adjacent years. **Appendix S6.** Database with underlying nutrient resorption data by species.
