# Supplementary material for: Mapping nutrient resorption efficiencies of subarctic cryptogams and seed plants onto the Tree of Life
Source: Ecol Evol. 2014 May 7;4(11):2217–27. doi: 10.1002/ece3.1079 (PMC4201435; doi:10.1002/ece3.1079)
Supplement: Supplementary file 1 [file ece30004-2217-sd1.doc]

**Appendix S1.** List of vascular plant and cryptogam species assessed for nutrient resorption efficiency.

Species are sorted according to clades (class and order where appropriate). RE_N_ was available for all species except *Eriophorum vaginatum*. Shaded species were not used in Figure 1 of the main manuscript. Marked with * are species for which RE_P_ was available. Species nomenclature follows Hill *et al.* (2006) for mosses except *Sphagnum*; Daniels & Eddy (1985) for *Sphagna*; Damsholt (2002) for liverworts; Santesson *et al.* (2004) for lichens; and Mossberg *et al.* (1992) for vascular plants.

| **Clade** | **Class** | **Order** | **Species** |
| --- | --- | --- | --- |
| Lichens | Lecanoromycetes | Lecanorales | *Cladonia uncialis* |
|  |  |  | *Cladonia amaurocraea* |
|  |  |  | *Cladonia arbuscula* |
|  |  |  | *Cladonia rangiferina* |
|  |  |  | *Cladonia stygia* |
|  |  |  | *Cladonia stellaris* |
|  |  |  | *Cetraria islandica* |
|  |  |  | *Cetrarialla delisei* |
|  |  |  | *Flavocetraria cucullata* |
|  |  |  | *Flavocetraria nivalis* |
|  |  |  | *Alectoria ochroleuca* |
|  |  |  | *Stereocaulon vesuvianum* |
|  |  |  | *Stereocaulon cf.grande* |
|  |  | Peltigerales | *Nephroma arcticum* |
|  |  |  | *Peltigera aphthosa* |
|  |  |  | *Solorina crocea* |
| Liverworts | Jungermanniopsida | Jungermanniales | *Nardia scalaris* |
|  |  |  | *Barbilophozia atlantica* |
|  |  |  | *Barbilophozia floerkii* |
|  |  |  | *Lophozia lycopodioides* |
|  |  | Ptilidiales | *Ptilidium ciliare* |
|  |  | Pelliales | *Pellia neesiana* |
|  | Marchantiopsida | Marchantiales | *Marchantia alpestris* |
| Mosses | Bryopsida |  | *Cinclidium stygium* |
|  |  |  | *Dicranum montanum* |
|  |  |  | *Dicranum fuscescens* |
|  |  |  | *Racomitrium microcarpon* |
|  |  |  | *Racomitrium fasciculare* |
|  |  |  | *Racomitrium lanuginosum* |
|  |  |  | *Hylocomium splendens* |
|  |  |  | *Pleurozium schreberi* |
|  |  |  | *Rhytidium rugosum* |
|  |  |  | *Tomenthypnum nitens* |
|  |  |  | *Aulacomnium palustre* |
|  |  |  | *Aulacomnium turgidum* |
|  |  |  | *Paludella squarrosa* |
|  | Polytrichopsida |  | *Polytrichastrum sexangulare* |
|  |  |  | *Polytrichum strictum* |
|  |  |  | *Polytrichum commune* |
|  | Sphagnopsida |  | *Sphagnum balticum* |
|  |  |  | *Sphagnum fuscum* |
| Mosses | Sphagnopsida |  | *Sphagnum riparium* |
|  |  |  | *Sphagnum teres* |
| Lycophytes |  |  | *Diphasiastrum complanatum* |
|  |  |  | *Lycopodium annotinum* |
| Monilophytes |  |  | *Equisetum sylvaticum* |
|  |  |  | *Gymnocarpium dryopteris* |
|  |  |  | *Matteuccia struthiopteris* |
| Conifers |  |  | *Juniperus communis* |
|  |  |  | *Picea* cf*. obovata* x *abies* |
|  |  |  | *Pinus sylvestris* |
| Eudicots |  |  | *Achillea millefolium* |
|  |  |  | *Alnus incana* |
|  |  |  | *Andromeda polifolia** |
|  |  |  | *Angelica sylvestris* |
|  |  |  | *Anthriscus sylvestris* |
|  |  |  | *Arctostaphylos alpinus* |
|  |  |  | *Astragalus alpinus* |
|  |  |  | *Astragalus frigidus* |
|  |  |  | *Bartsia alpina* |
|  |  |  | *Betula nana** |
|  |  |  | *Betula pubescens* |
|  |  |  | *Bistorta vivipara* |
|  |  |  | *Caltha palustris* |
|  |  |  | *Cassiope tetragona* |
|  |  |  | *Cornus suecica* |
|  |  |  | *Dryas octopetala* |
|  |  |  | *Empetrum nigrum** |
|  |  |  | *Epilobium angustifolium* |
|  |  |  | *Filipendula ulmaria* |
|  |  |  | *Geranium sylvaticum* |
|  |  |  | *Lathyrus pratensis* |
|  |  |  | *Orthilia secunda* |
|  |  |  | *Pedicularis hirsuta* |
|  |  |  | *Pedicularis lapponica* |
|  |  |  | *Pedicularis sceptrum-carolinum* |
|  |  |  | *Populus tremula* |
|  |  |  | *Rhodiola rosea* |
|  |  |  | *Rhododendron lapponicum* |
|  |  |  | *Ribes spicatum* |
|  |  |  | *Rubus chamaemorus** |
|  |  |  | *Rubus saxatilis* |
|  |  |  | *Rumex obtusifolius* |
|  |  |  | *Salix herbacea* |
|  |  |  | *Salix lapponum* |
|  |  |  | *Salix myrsinites* |
|  |  |  | *Salix reticulata* |
|  |  |  | *Solidago virgaurea* |
|  |  |  | *Sorbus aucuparia* |
|  |  |  | *Tanacetum vulgare* |
|  |  |  | *Trifolium pratense* |
|  |  |  | *Trollius europaeus* |
| Eudicots |  |  | *Vaccinium myrtillus* |
|  |  |  | *Vaccinium uliginosum** |
|  |  |  | *Vaccinium vitis-idaea* |
|  |  |  | *Veronica alpina* |
|  |  |  | *Vicia cracca* |
| Monocots |  |  | *Calamagrostis lapponica* |
|  |  |  | *Carex capitata* |
|  |  |  | *Carex rostrata* |
|  |  |  | *Carex saxatilis* |
|  |  |  | *Carex vaginata* |
|  |  |  | *Deschampsia cespitosa* |
|  |  |  | *Elytrigia repens* |
|  |  |  | *Eriophorum angustifolium* |
|  |  |  | *Eriophorum vaginatum* |
|  |  |  | *Juncus arcticus* |
|  |  |  | *Juncus trifidus* |
|  |  |  | *Luzula multiflora* |
|  |  |  | *Phleum alpinum* |

**References**

Damsholt, K. (2002) Illustrated Flora of Nordic Liverworts and Hornworts. Nordic Bryological Society, Lund.

Daniels, R.E. & Eddy, A. (1985) Handbook of European *Sphagna*. Institute of Terrestrial Ecology, Huntingdon.

Hill, M.O., Bell, N., Bruggeman-Nannenga, M.A., Brugués, M.*, et al.* (2006) An annotated checklist of the mosses of Europe and Macaronesia. *Journal of Bryology*, **28**, 198-267.

Mossberg, B., Stenberg, L. & Ericsson, S. (1992) Den Nordiska Floran. Wahlström and Widestrand, Stockholm.

Santesson, R., Moberg, R., Nordin, A., Tønsberg, T.*, et al.* (2004) Lichen-forming and Lichenicolous Fungi of Fennoscandia. Museum of Evolution, Uppsala University, Uppsala.

**Appendix S2.** Early versus late RE in lichens and mosses.

Early RE of both N and P in mosses was always higher compared to late RE (Table 1), which even took on negative values (Fig. 1). These might indicate translocation of nutrients into brown moss tissue. Based on upward and downward movement of ^14^C in the moss shoot, Hakala & Sewón (1992) suggested that senescent moss parts function as an energy store. Early RE in lichens was significantly higher for RE_N%_ while RE_Nsr_ showed a trend for the same pattern. Early versus late RE_P_ was not significantly different for lichens.

Table 1: Early versus late RE_N%_, RE_Nsr_, RE_P%_ and RE_Psr_ of mosses and lichens (*n* = 15; the outliers late RE of *Cetraria islandica* and early RE of *Stereocaulon* cf. *grande* were excluded). Significant *P*-values are marked with bold letters

| **Clade** | **Source** | **d.f.** | ***F*** | ***P*** |
| --- | --- | --- | --- | --- |
| Moss | RE_N%_ | 1 | 18.24 | **< 0.001** |
|  | RE_Nsr_ | 1 | 15.55 | **0.001** |
|  | RE_P%_ | 1 | 6.83 | **0.014** |
|  | RE_Psr_ | 1 | 7.89 | **0.010*** |
| Lichen | RE_N%_ | 1 | 25.73 | **< 0.001** |
|  | RE_Nsr_ | 1 | 3.61 | 0.068 |
|  | RE_P%_ | 1 | 0.16 | 0.69 |
|  | RE_Psr_ | 1 | 0.11 | 0.74 |

* Ranked

Fig. 1. Early versus late RE_N%_, RE_Nsr_, RE_P%_ and RE_Psr_ for mosses and lichens (Tukey, *n* = 13-15).

Independent of time, non-N_2_-fixing lichens showed significantly higher RE_P_ (40-50%) compared to N_2_-fixing lichens (3-12%) while the interaction of time x N_2_-fixation was not significant (Table 2). Possible explanations for this finding are given in the Discussion (see main manuscript).

Table 2. Comparison of N_2_-fixation and time of resorption on RE_P%_ and RE_Psr_ of lichens (n = 15; the outliers late RE of *Cetraria islandica* and early RE of *Stereocaulon* cf. *grande* were excluded). Significant *P*-values are marked with bold letters

| **Variable** | **Source** | **d.f.** | ***F*** | ***P*** |
| --- | --- | --- | --- | --- |
| RE_P%_ | N_2_-fixation | 1 | 5.78 | **0.024** |
|  | Time | 1 | 0.23 | 0.63 |
|  | N_2_-fixation x time | 1 | 0.39 | 0.54 |
| RE_Psr_ | N_2_-fixation | 1 | 8.71 | **0.007** |
|  | Time | 1 | 0.67 | 0.42 |
|  | N_2_-fixation x time | 1 | 0.51 | 0.48 |

**References**

Hakala, K. & Sewón, P. (1992) Reserve lipid accumulation and translocation o^f^ 14C in the photosynthetically active and senescent shoot parts o*f Dicranum elongat*um*. Physiologia Plantar*um**,** 85, 111-119.

**Appendix S3.** Prediction of cellulose content inferred from infrared spectra.

Table 1. Calibration and prediction of cellulose [%] from infrared spectra (PLS-R, *n* = 14)

|  | **Cellulose** |
| --- | --- |
| N | 14 |
| No. of PCs* | 6 |
| R^2^_Cal._† | 0.98 |
| R^2^_Pred_. | 0.91 |
| RMSE_Cal._‡ | 0.95 |
| RMSE_Pred_. | 2.02 |
| Slope_Cal_. | 0.98 |
| Slope_Pred_. | 0.99 |
| Intercept_Cal_. | 0.41 |
| Intercept_Pred_. | 0.08 |

* PC: Principal component

† Cal.or Pred.: Calibration or prediction

‡ RMSE: Root mean square error

Fig. 1. FTIR-derived vs. wet chemical measurements cellulose (*n* = 7; the outlier was excluded).

**Appendix S4.** Comparison of RE calibration approaches.

Although the pattern for most calibration methods seemed to be similar since none of the interaction terms was significant (Table 1), variation was especially high in RE_NCa_ (Fig. 1). The use of RE_NCa_ for mosses is problematic since Ca is known to either accumulate in old moss tissue (Vitt & Pakarinen 1987), move about mosses (Wells & Brown 1996) or even show no differences to slight decreases in young versus older tissue (Malmer 1993). It therefore provides an unreliable basis in contrast to this method used for vascular plants (Soudzilovska*ia et a*l. 2007). Furthermore, in lichens, Ca may occur in trapped particles or as Ca oxalate but is also bound extracellularly (Brown 1987). As older material decomposes, trapped material or Ca oxalate might be lost from the tissue or decomposition of material might create additional exchange sites by increasing the tissue surface. Thus, it seems unsure whether Ca would provide a safe basis for RE.

**Table 1.** Comparison of measurement type of RE_N_ at clade, class and order level (ranked; *n* = 3-20)

| **Taxonomical level** | **Source** | **d.f.** | ***F*** | ***P*** |
| --- | --- | --- | --- | --- |
| Clade | Clade | 2 | 8.94 | < 0.001 |
|  | Method | 2 | 0.01 | 0.99 |
|  | Clade x method | 4 | 0.39 | 0.82 |
| Class | Class | 2 | 6.84 | 0.002 |
|  | Method | 2 | 0.30 | 0.74 |
|  | Class x method | 4 | 0.81 | 0.53 |
| Order | Order | 1 | 1.22 | 0.28 |
|  | Method | 2 | 0.64 | 0.54 |
|  | Order x method | 2 | 1.77 | 0.19 |

Fig. 1. RE_N%_, RE_NCa_ and RE_Nsr_ across and within clades. Different letters indicate significance at *P* < 0.05 (Tukey, n = 3-20).

**References**

Brown, D.H. (1987) The location of mineral elements in lichens; implications for metabolism. Progress and Problems in Lichenology in the Eighties (ed E. Peveling), pp. 361-375. J. Cramer, Berlin.

Malmer, N. (1993) Mineral nutrients in vegetation and surface layers of *Sphagnum*-dominated peat-forming systems. *Advances in Bryology*, **5**, 223-248.

Soudzilovskaia, N.A., Onipchenko, V.G., Cornelissen, J.H.C. & Aerts, R. (2007) Effects of fertilisation and irrigation on 'foliar afterlife' in alpine tundra. *Journal of Vegetation Science*, **18**, 755-766.

Vitt, D.H. & Pakarinen, P. (1987) The bryophyte vegetation, production and organic components of Truelove Lowland. Truelove Lowland, Devon Island, A High Arctic Ecosystem (ed L.C. Bliss), pp. 225-244. The University of Alberta Press, Edmonton.

Wells, J.M. & Brown, D.H. (1996) Mineral nutrient recycling within shoots of the moss *Rhytidiadelphus squarrosus* in relation to growth. *Journal of Bryology*, **19**, 1-17.

**Appendix S5.** Vascular plant [N_senesced_] and [N_senesced_]/[cellulose] of adjacent years.

Comparison of [N_senesced_] and [N_senesced_]/[cellulose] of adjacent years (1998 versus 1999, n=15), based on vascular plant data from Quested *et al.* (2003).

**References**

Quested, H.M., Cornelissen, J.H.C., Press, M.C., Callaghan, T.*V., et a*l. (2003) Decomposition of sub-arctic plants with differing nitrogen economies: a functional role for hemiparasites*. Ecolo*gy**,** 84, 3209-3221.
